# Transcriptional regulation of early embryo development in the model legume *Medicago truncatula*

**DOI:** 10.1007/s00299-013-1535-x

**Published:** 2013-11-22

**Authors:** Sergey Kurdyukov, Youhong Song, Michael B. Sheahan, Ray J. Rose

**Affiliations:** 1 Australian Research Council Centre of Excellence for Integrative Legume Research, School of Environmental and Life Sciences, The University of Newcastle, Callaghan, NSW 2308 Australia; 2 Present Address: Kolling Institute of Medical Research, Kolling Building, Royal North Shore Hospital, St Leonards, NSW 2065 Australia

**Keywords:** *Medicago truncatula*, Embryogenesis, Ovules, Seed development, Transcription factors, Homeobox genes

## Abstract

*****Key message***:**

**Spatial and temporal expression of co-expressed transcription factors provide a framework to investigate the integrated control of embryo size, vascularisation, meristem development and onset of seed filling in early embryogenesis of** ***Medicago truncatula.***

**Abstract:**

Cultivated legumes account for more than a quarter of primary crop production worldwide. The protein- and oil-rich seed of cultivated legumes provides around one-third of the protein in the average human diet, with soybeans (*Glycine max* (L.) Merr) being the single largest source of vegetable oil. Despite their critical importance to human and animal nutrition, we lack an understanding of how early seed development in legumes is orchestrated at the transcriptional level. We developed a method to isolate ovules from the model legume, *Medicago truncatula* Gaertn, at specific stages of embryogenesis, on the basis of flower and pod morphology. Using these isolated ovules we profiled the expression of candidate homeobox, AP2 domain and B3 domain-containing transcription factors. These genes were identified by available information and sequence homology, and five distinctive patterns of transcription were found that correlated with specific stages of early seed growth and development. Co-expression of some genes could be related to common regulatory sequences in the promoter or 3′-UTR regions. These expression patterns were also related to the expression of B3-domain transcription factors important in seed filling (MtFUS3-like and MtABI3-like). Localisation of gene expression by promoter-GUS fusions or in situ hybridisation aided understanding of the role of the transcription factors. This study provides a framework to enhance the understanding of the integrated transcriptional regulation of legume embryo growth and development and seed filling.

**Electronic supplementary material:**

The online version of this article (doi:10.1007/s00299-013-1535-x) contains supplementary material, which is available to authorized users.

## Introduction

Fertilisation of the egg and central cell within an ovule by the male gametes triggers a process that produces a diploid embryo and triploid endosperm, and ultimately leads to the development of a seed. After fertilisation of the egg, the zygote undergoes an asymmetric cell division producing a two-cell embryo polarised along the apical–basal axis (Goldberg et al. 1994; Wang et al. 2012). In the model legume, *Medicago truncatula*, the program of embryonic development nears completion by around 8 days after anthesis, at which point a fully formed embryonic body-plan and all specialised meristematic tissues are evident (Wang et al. 2012). Establishing an embryonic body-plan requires appropriate cell differentiation, which in turn necessitates a coordinated regulation of gene expression.

In Arabidopsis (*Arabidopsis thaliana*), a number of transcription factors act exclusively during embryogenesis, with many exhibiting precise spatiotemporal expression patterns (Park and Harada 2008; Le et al. 2010). Such specialised expression is necessary to establish axes of polarity, which in turn determine the correct placement and development of organs and tissues such as the vasculature, meristems and epidermis. While there are considerable data concerning the genetic regulation of early embryogenesis in Arabidopsis, with the exception of soybean (Le et al. 2007), little is known of analogous processes in legume species. Legume embryos display a wide range of forms and their protein- and oil-rich cotyledons supply much of the world’s protein and plant oil needs (Graham and Vance 2003; Le et al. 2007). With its small-sized (500 Mb), diploid genome and short life cycle, *M. truncatula* (along with *Lotus japonicus* L.) has become the primary experimental model for legumes, being used to study nodulation, but also seed and embryo development (Cook 1999; Rose 2008; Cannon et al. 2009; Thompson et al. 2009; Rose et al. 2010). Moreover, *M. truncatula* is phylogenetically close to the most economically important, but less experimentally tractable legumes and displays large-scale synteny with other legumes (Choi et al. 2004; Cannon et al. 2006; Young and Udvardi 2009). Thus, knowledge generated in *M. truncatula* should be readily transferrable to important crop species, making it an important model for forage and grain legumes.

Substantive genomic resources exist for *M. truncatula* (Young and Udvardi 2009). For example, publicly available Affymetrix Gene Chip data for *M. truncatula* have been abridged in the interactive *M. truncatula* Gene Expression Atlas (MtGEA; Benedito et al. 2008). While the MtGEA summarises information on transcription factors involved in the latter stages of seed development, the early stages (<10 days after anthesis) corresponding to embryogenesis are unrepresented by this resource. Studies by Verdier et al. (2008) investigating transcriptional regulation of seed filling in *M. truncatula* were carried out from 10 days after pollination. Obtaining sufficient embryonic material in *M. truncatula* has hindered investigations into the genetic regulation of early seed development in this species. The acquisition of temporal and spatial expression data and the identification of *cis*-elements common to co-expressed genes can assist in making informed predictions of gene function.

We recently completed an investigation into the developmental biology of embryogenesis in *M. truncatula* from embryo sac to embryo maturation and oil and protein body formation (Wang et al. 2012). Here, we sought to provide a standardised method of collecting ovules at the same developmental stage to collect a uniform population of embryonic material to profile the expression of candidate transcriptional regulators of embryogenesis. By interrogating databases of expressed sequence tags and genomic sequences, we identified genes with homology to transcription factors active in flowers or seeds and, therefore, possibly involved in early seed development. In choosing the transcription factors for our analysis, we also considered their regulatory potential. Profiling the expression of 19 candidate genes by quantitative real-time PCR (qRT-PCR) revealed distinctive patterns of transcription during early seed development. We also related the timing of the expression of these genes to the transcription factor MtSERF1, essential for somatic embryogenesis in *M. truncatula* (Mantiri et al. 2008), and to genes related to seed filling. For selected candidates, we also performed RNA in situ hybridisation and promoter-GUS experiments, which revealed embryo- and tissue-specific expression patterns. By analysing sequences outside the open reading frame of co-expressed genes, we discovered motifs with shared sequence similarity. Our data complement studies of seed embryo development in *M. truncatula* (Wang et al. 2012), providing a framework for understanding how transcriptional control orchestrates legume seed development.

## Materials and methods

### Plant growth

Plant materials were obtained from glasshouse-grown plants of *M. truncatula* Gaertn cv. Jemalong. The glasshouse had a 14-h photoperiod and 23/19 °C day/night temperature regime.

### Ovule collection and RNA isolation

**Fig. 1:**
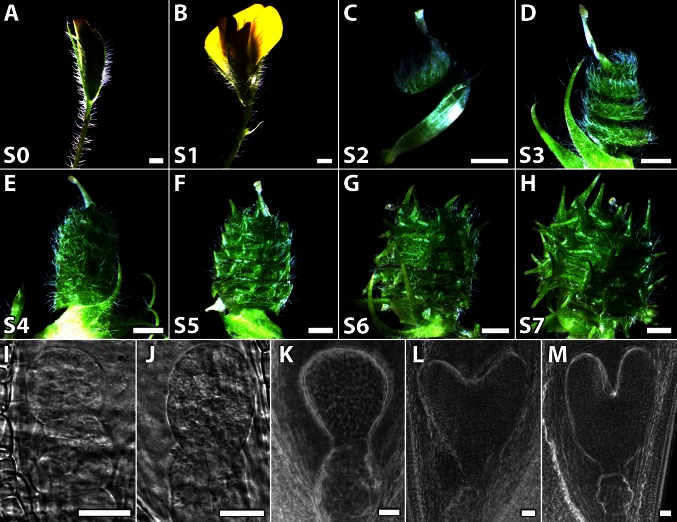
Pods of Medicago collected for analysis and corresponding stages of embryogenesis. **a**–**h** Images of flowers and pods (with petals removed) used to isolate RNA for qRT-PCR. **a** Stage 0: flower bud at 2 days before anthesis. **b** Stage 1: flower at anthesis. **c** Stage 2: early pod with one complete spiral. **d** Stage 3: pod with three complete spirals corresponding to early embryo development. **e** Stage 4: pod with five complete spirals and spine initials corresponding to early globular embryos. **f** Stage 5: pod with six spirals and immature spines corresponding to late globular embryos. **g** Stage 6: pod with six spirals and elongated, maturing spines corresponding to heart-stage embryos. **h** Stage 7: Pod with six spirals, mature spines and increased girth corresponding to torpedo-stage embryos. **i**–**m** Zygotic embryos as visualised in ovules isolated from the middle portion of pods corresponding to stages S3–S7. **i** 8–16 cell-stage embryo. **j** Early globular-stage embryo. **k** Late globular-stage embryo. **l** Heart-stage embryo. **m** Torpedo-stage embryo. *Bars* 2 mm (**a**–**h**) and 25 μm (**i**–**m**)

Ovules were collected from flower buds (2 days before anthesis, as determined by bud size and petal colour; Stage 0), flowers at anthesis (Stage 1) and from pods at six developmental stages from 2–7 days after anthesis (Fig. 1; Table S1). For each of two independent experiments, ovules were collected at each developmental stage. To minimise variation, ovules were collected solely from the middle of each pod. Ovules were isolated from pods using a stereo dissection microscope (Leica, MZFLIII). In addition, only a small number of pods were collected at a time and kept on ice for not longer than 1 h. Ovules were stored at −80 °C until sufficient numbers were obtained. Ovules were suspended in ice-cold RNA lysis/binding solution from an RNAqueous-4PCR RNA isolation kit (Ambion). After isolation, the ovules were centrifuged and the supernatant transferred to a fresh tube. The pellet was immediately frozen in liquid nitrogen and homogenised with a plastic micropestle. Lysis/binding solution was returned to the homogenate and vortexed for 10 min. Cell debris was removed by centrifugation and RNA isolated (with DNase treatment) according to the manufacturer’s instructions (Ambion). The methods for seed isolation and RNA extraction for *MtFUS3*-*like* and *MtABI3*-*like* gene expression during maturation were as described (Wang et al. 2012) and three replicates were used.

### Selection of transcription factors

The transcription factors selected were likely to be involved in different stages of early embryo development (Tables S2 and S3). We had identified these transcription factors in somatic embryogenesis studies in *M. truncatula* (Mantiri et al. 2008; Chen et al. 2009) or where information was available from *M. truncatula* (Di Giacomo et al. 2008; Imin et al. 2007). We also identified transcription factors up-regulated in flowers and developing seeds in the MtGEA and from Arabidopsis using microarray data maintained in the Arabidopsis gene expression atlas (http://www.ebi.ac.uk/gxa). BLAST searches (tblastx and blastx) were used to find homologues to these transcription factors in *M. truncatula* EST collections (DFCI Medicago Gene Index; http://compbio.dfci.harvard.edu/tgi) or from genomic sequences (GenBank; http://www.ncbi.nlm.nih.gov). The corresponding gene loci and protein sequences were obtained from the Mt3.5 genome release (http://www.medicagohapmap.org), or where unavailable, they were predicted directly using FGENESH (http://www.softberry.com). Full-length amino acid sequences of homologues from Arabidopsis, *M. truncatula* and rice (*Oryza sativa*) were then aligned using ClustalX 2.0.10 (Larkin et al. 2007). Phylograms (Supplementary Fig. 1A) were constructed from aligned sequences and drawn using the MEGA 5 program (Tamura et al. 2011) or (Supplementary Figs. 1B and 1C) were constructed from aligned sequences using the protein maximum likelihood, proml, programme in PHYLIP [Phylogeny Inference Package Version 3.69; (Felsenstein 1989)] and drawn with Dendroscope (Huson et al. 2007).

### Gene expression analysis by quantitative real-time PCR

Synthesis of cDNA from 4 μg of total RNA was performed using a Superscript III kit (Invitrogen) following the manufacturer’s instructions. Gene expression was analysed by quantitative real-time PCR (qRT-PCR) using a RotorGene-Q (Qiagen). PCR reactions were carried out using glyceraldehyde 3-phosphate dehydrogenase (GAPDH; Mt8g109660) as a reference gene. GAPDH is a suitable reference gene for *M. truncatula* seed development based on geNORM software (Verdier et al. 2008), and our previous microarray and qRT-PCR studies (Mantiri et al. 2008). PCR master mixes were prepared with Platinum Taq (Invitrogen) using the provided buffer supplemented with 1.5 μM SYTO9 (Invitrogen), 3 mM dNTPs and 0.4 μM of each primer. The qRT-PCR cycling conditions comprised an initial denaturation at 95 °C for 2 min followed by 40 cycles of 95 °C for 10 s, 60 °C for 30 s and 72 °C for 30 s. Dissociation analysis (0.5 °C step) was performed in every run and products checked by gel electrophoresis to ensure product uniformity. For each gene analysed, two biological and three technical repetitions were performed. Data analyses were performed using Q-Gene software (http://download.gene-quantification.info/; Muller et al. 2002; Simon 2003). Q gene software uses mean normalised data and the ΔΔ*C*_T_ method to calculate relative expression (the calibrator was the lowest expression point for the gene investigated) and standard errors. Primers used for qRT-PCR are listed in Table S2 and amplification efficiency based on serial dilution was greater than 90 %.

### Construction and analysis of pMtWOX9-like::GUS fusion in somatic embryos

The promoter of a *MtWOX9*-*like* gene was amplified from *M. truncatula* genomic DNA by the primers 5′-CACCTTTAGCGCGCACCGTATTG-3′ and 5′-ACAGTGTATGACATAAAAACGGAAGAT-3′ to give 1.65 kb of promoter sequence ending at the transcription start site 148 bp upstream of the ATG start codon. The PCR amplicon was cloned into a pCR8/GW/TOPO entry vector (Invitrogen) and the orientation of the sequence confirmed by sequencing. The entry clone was transferred to the Gateway destination vector, pHGWFS7 (Karimi et al. 2005), using LR recombination and the resulting construct transformed into *Agrobacterium tumefaciens* [strain AGL1; (Lazo et al. 1991)] as described (Nolan et al. 2009). Transformation and culture of leaf explants was as described (Nolan et al. 2009). For GUS localisation studies, twenty calli from four independent transformations were cultured on P4:10:4 medium for 6 weeks and stained for GUS activity (Nolan et al. 2009).

### RNA in situ hybridisation

Sequence-specific probes (as determined by blast checks) for in situ hybridisation were prepared by PCR from pooled cDNA using the primers 5′-CAATTCAATGTTGCACCAAACTA-3′ and 5′-ATTATCCACTGCAGTTCCAGCTA-3′ for *MtABI3*-*like* (TC162720); 5′-TGGAATGGAATGGAAACACAAGAAGTTT-3′ and 5′-GGTGATAGTTATAAGGTACAGCAGCA-3′ for *MtLMI1*-*like* (Mt1g073710). Primer sequences for the *MtWUS* (*MtWUSCHEL*) probe and the RNA in situ hybridisation procedure were as described (Chen et al. 2009).

### Microscopy

Ovules from each developmental stage for Fig. 1 were cleared using Hoyer’s solution (Nolan et al. 2009). For in situ localisation, ovules were fixed in 4 % formaldehyde and processed as described (Chen et al. 2009). Sectioned material was examined using a compound microscope (Zeiss; Axiophot), while cleared ovules and GUS-stained embryos were visualised with a stereo dissection microscope (MZFLIII). Images were captured with a digital camera (Zeiss, AxioCam HRc).

### In silico analysis

Identification of common elements in the promoters and 3′-untranslated regions (3′-UTRs) of co-expressed genes was performed using MClip with default cut-off of 1e^−3^ [(Frickey and Weiller 2007); http://bioinfoserver.rsbs.anu.edu.au/utils/mclip]. Sequences up to 4 kb upstream of the start codon for promoters and 1 kb downstream of the stop codon for 3′-UTRs were aligned and then analysed by MClip.

## Results

### Linking the early stages of embryogenesis in *M*. *truncatula* to pod morphology

We defined eight stages of early seed development (S0-S7; Table S1)—flower buds at 2 days before anthesis (S0), flowers at anthesis (S1) and six stages of pod development (S2–S7) that encompassed early embryogenesis (8–16 cell embryos) until the torpedo stage of embryogenesis (Fig. 1). The approximate timeframe, relative to anthesis, for collecting flowers and pods is given in Table S1. We found that embryo development varied with the time after anthesis, even though plants were of the same age and grown under identical conditions. However, by isolating ovule populations from pods defined by morphological criteria, we could isolate uniform populations of ovules containing embryos at a similar developmental stage. The criteria we used to define pod morphology were the number of pod spirals, developmental stage of pod spines and pod girth (Fig. 1). We distinguished pod stages S2–S4 primarily by the number of spirals present, with a single complete spiral in S2 pods, three in S3 pods and five in S4 pods. Pods at the S4 stage also exhibited spine initials (Fig. 1c–e). The presence of six complete spirals and immature spines whose length did not exceed the width of an individual pod spiral marked the S5 stage (Fig. 1f). Spines of S6 pods were longer than the width of an individual pod spiral and slightly bent, while S7 pods possessed thicker spines again and an increased pod girth relative to S6 pods (Fig. 1g, h). We did, however, find it essential to measure the time elapsed between S6 and S7 pods, as S7 pods were often indistinguishable from older pods (Table S1). The developmental stage of embryos corresponding to ovules isolated from S3 to S7 pods is shown in Fig. 1i–m. The uniformity of ovule development was confirmed by routinely examining embryos isolated from randomly selected ovules. It is difficult to identify embryos at the 1–4 cell stage and during sectioning; the embryo is often lost as it is not well attached to surrounding tissue. Thus, ovules of a specific developmental stage can be isolated, without the need for embryo isolation or sectioning and laser capture microdissection.

### Selection of transcription factors for expression profiling

**Fig. 2:**
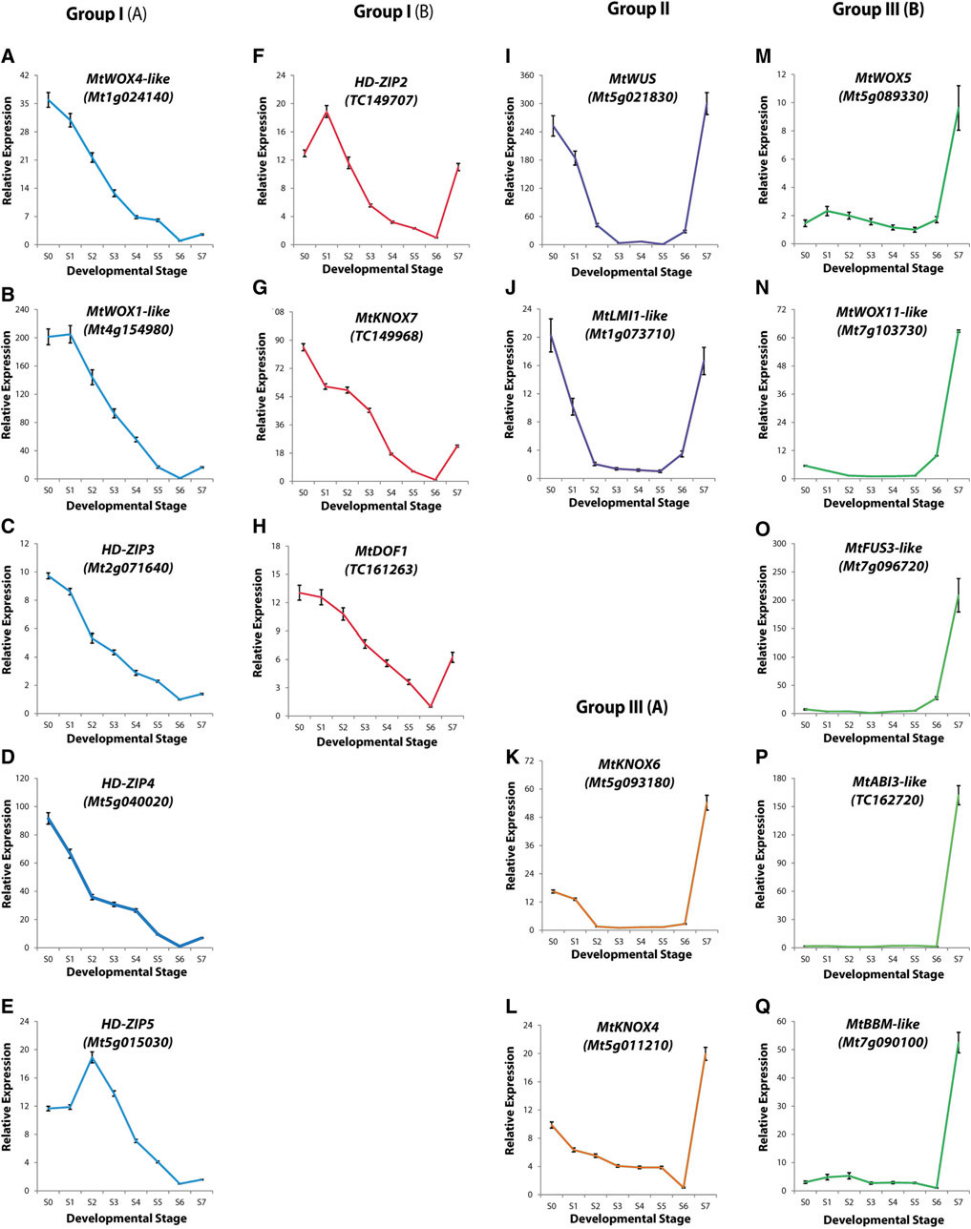
Expression profile of 17 transcription factors in Groups I, II and III during ovule and embryo development. RNA was extracted from ovules at eight developmental stages (S0–S7) as represented in Fig. 1, with genes grouped on the basis of expression pattern. **a**–**e** Group I(A): continuously down-regulated during ovule and embryo development. **f**–**h** Group I(B): down-regulated during ovule and embryo development and then up-regulation commenced in torpedo-stage embryos. **i**, **j** Group II: high expression in ovule development and very early embryogenesis, inactive during early embryogenesis and markedly up-regulated during heart- and torpedo-stage embryogenesis. **k**, **l** Group III(A): some expression in ovule development and very early embryogenesis then down-regulated until marked increase in expression in torpedo-stage embryos. **m**–**q** Group III(B): high expression only in late heart- and torpedo-stage embryogenesis. Genes *colour*-coded to indicate members common to a transcription factor group. Standard errors indicated

Among the 14 homeobox genes we selected, six belong to the *WUSCHEL*/*WUSCHEL*-*RELATED HOMEOBOX* (*WUS*/*WOX*) gene family, five to the *HOMEOBOX LEUCINE ZIPPER* (*HD*-*ZIP*) gene family and three to the *KNOTTED1*-*LIKE HOMEOBOX* (*KNOX*) gene family. In addition to the homeobox genes, we selected a *DNA*-*BINDING WITH ONE ZINC FINGER* (DOF) transcription factor, *BABY BOOM* which contains two AP2/ERF DNA-binding domains, *SOMATIC EMBRYO-RELATED FACTOR 1* (*MtSERF1*) which contains a single AP2/ERF domain and two genes from the *B3 DOMAIN*-*CONTAINING* transcription factor family (*FUS3*-*like* and *ABI3*-*like*). The loci for these genes in the *M. truncatula* genome, corresponding to the closest Arabidopsis homologue and Affymetrix *M. truncatula* GeneChip probesets used to examine gene expression are listed in Table S3. Table S3 indicates what was known of the expression of these genes in *Medicago truncatula*, based predominantly on the MtGEA. Using qRT-PCR to profile the expression of these transcription factors, we identified five overall types of expression patterns (Fig. 2).

### Group I genes

Group I(A) genes comprised those showing decreased expression during ovule and embryo development (S0-S7; Fig. 2a–e). The *MtWOX4*-*like* (Fig. 2a), *MtWOX1*-*like* (Fig. 2b), *HD*-*ZIP3* (Fig. 2c) and *HD*-*ZIP4* (Fig. 2d) had a peak expression at S0, 2 days before anthesis at the ovule development stage. The *HD*-*ZIP5* gene had high expression at the ovule development stages (S0 and S1), but peaked at the earliest stage of embryogenesis, S2, before declining (Fig. 2e). The three Group I(B) genes [*HD*-*ZIP2* (Fig. 2f), *MtKNOX7* (Fig. 2g) and *MtDOF1* (Fig. 2h)] had similar expression to the Group 1(A) genes during ovule and early embryo development; but at the torpedo stage (S7; Fig. 2f–h), expression started to increase again.

### Group II genes

**Fig. 3:**
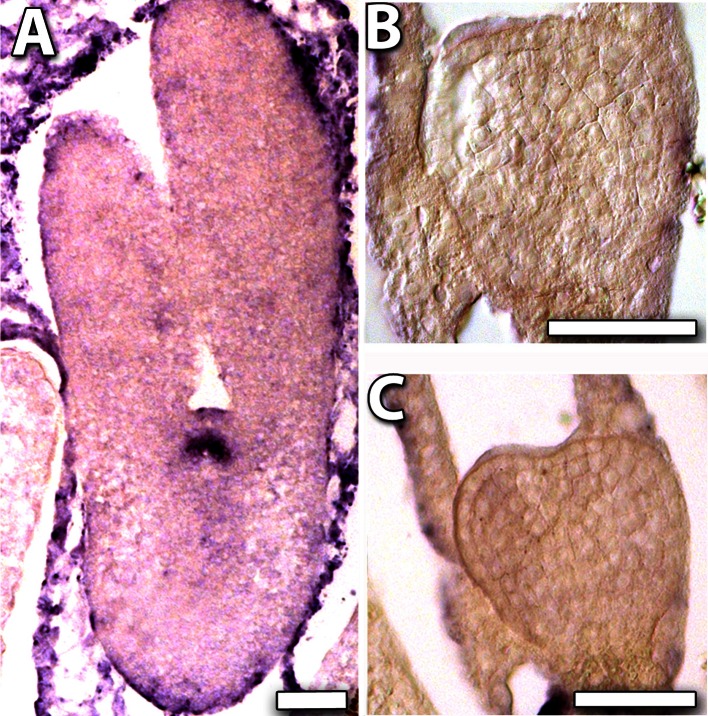
Expression localisation for *MtWUS* by in situ hybridisation. **a** Torpedo-stage embryo. **b** Globular-stage and **c** late globular-stage transitioning to heart-stage embryo show no signal. *Bars* 50 μm

The Group II genes, *MtWUS* (Fig. 2i) and *MtLMI1*-*like* (Fig. 2j) had a “U” shaped profile. Quite clearly there were two high points of expression at S0 and S7, in the ovule development and torpedo stages. In the case of these two genes, we carried out in situ hybridisation studies. In torpedo-stage embryos *MtWUS* was strongly expressed in the organiser region (Fig. 3a), consistent with previous studies (Chen et al. 2009); although there was some background on the embryo edge. There was no in situ hybridisation in the earlier stage embryos (Fig. 3b, c), consistent with qRT-PCR results. *WUS* was expressed in ovule development (Fig. 2i) as occurs in Arabidopsis (Groß-Hardt et al. 2002).

**Fig. 4:**
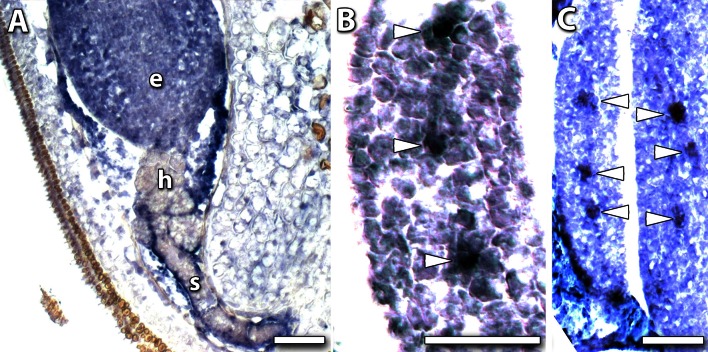
Expression localisation of *MtLMI*-*like* genes. In situ hybridisation against the *MtLMI1*-*like* (Mt1g073710) gene expression. **a** Early torpedo-stage embryo (e) with no signal in the hypophysis (h) or suspensor (s). **b**, **c** Transverse sections of cotyledons in a torpedo-stage embryo showing a concentration of signal in forming vascular tissue (*arrowheads*). *Bars* 50 μm

**Fig. 5:**
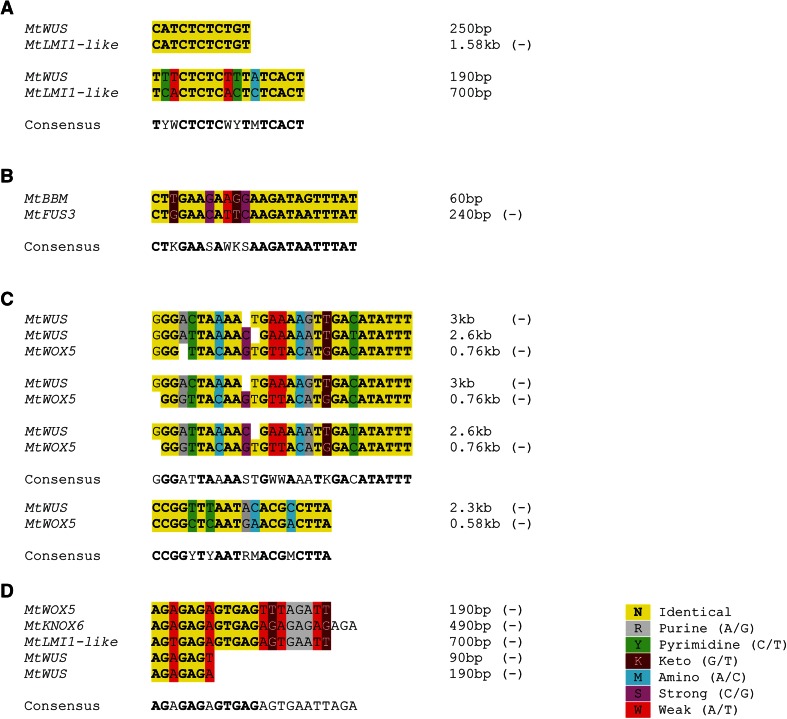
Sequence elements in co-expressed and orthologous genes. **a** Region upstream of the *MtWUS* and *MtLMI1*-*like* start codon contains two common sequence elements. **b** A common sequence element in the 3′-UTR of *MtBBM* and *MtFUS3*-*like.* **c** Conserved sequence elements in the region upstream of the *MtWUS* and *MtWOX5* start codon. Upper alignment for the first motif represents the two individual motifs in the *MtWUS* promoter aligned to the *MtWOX5* promoter motif, whereas subsequent alignments show individual *MtWUS* motifs aligned to the *MtWOX5* motif. **d** Upstream regions of the co-expressed *MtWOX5*, *MtKNOX6*, *MtLMI1*-*like* and *MtWUS* genes contain a GAGA-like *cis*-element originally identified in soybean

The *M. truncatula* homologue of Arabidopsis *LATE MERISTEM IDENTITY1* (*MtLMI1*-*like*) encodes a Class I HD-ZIP protein with an expression profile (Fig. 2j) that closely resembles *MtWUS* (Fig. 2i). RNA in situ hybridisation revealed that expression of *MtLMI1*-*like* localised throughout the whole embryo at the torpedo stage with a notable absence of expression in the hypophysis and suspensor (Fig. 4a). The expression of *MtLMI1*-*like* was, however, particularly concentrated in the vascular procambium in torpedo-stage embryos (Fig. 4b, c). *MtWUS* and *MtLMI1*-*like* contained two common promoter elements—one consisting of 11 bp of identical sequence, and the other 17 bp of interrupted sequence with an overall identity of 71 % (Fig. 5a).

### Group III genes

Group III(A) genes were highly expressed during the torpedo stage (S7; Fig. 2k, l), but with moderate expression in the ovule development stage. Group III(B) genes dramatically increased expression at the torpedo stage (Fig. 2m–q). These genes are known to be associated with both embryo development and seed filling (Boutilier et al. 2002; Haecker et al. 2004; Verdier and Thompson 2008). Despite their distinctive cellular functions, *MtBBM* and *MtFUS3*-*like* exhibited a similar expression profile. Interestingly, both genes contain a 23-bp motif within their 3′-UTR that is 78 % identical and contains a 12-bp stretch of complete identity (Fig. 5b). It is possible the motif represents a miRNA target, but it is more likely to be a *cis*-element recognised by a DNA-binding protein, as it is present on the sense strand in *MtBBM* yet on the antisense strand in *MtFUS3*-*like*.

Although there are no previous reports of *cis*-elements common to *MtWUS* and *MtWOX5* promoters, we found relatively long stretches (28 and 20 bp) of homologous sequence in these promoters from *M. truncatula*, which shared 61 and 75 % identity, respectively (Fig. 5c). Furthermore, a GAGA-like element identified in soybean (Sangwan and O’Brian 2002) was present in the promoters of *MtWUS* and *MtLM1* in expression Group II, *MtKNOX6* in expression Group III(A) and *MtWOX5* in expression Group III(B) (Fig. 5d). These four genes were all strongly up-regulated at the torpedo stage.

**Fig. 6:**
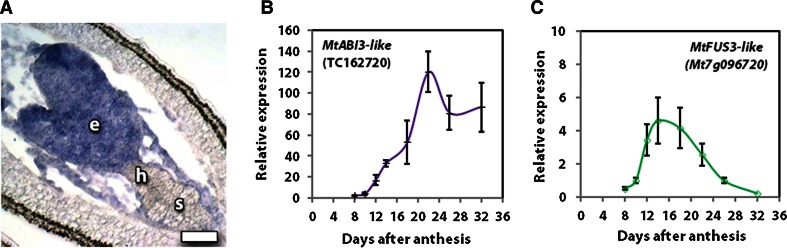
Expression of *ABI3*-*like* and *FUS3*-*like* genes. In situ hybridisation (**a**) of *ABI3* shows expression throughout the torpedo-stage S7 embryo with signal localised to the embryo, (e) but absent from the hypophysis (h) and suspensor (s). Expression profile during cotyledon development and seed filling is shown for *ABI3* (**b**) and *FUS3* (**c**). *Bar* 50 μm

In legume seed, a major interest is in the regulation of accumulation of storage products in the developing cotyledons. The expression of the ABI3 transcription factor is associated with storage accumulation and controlled desiccation (Verdier and Thompson 2008). In situ hybridisation showed expression throughout the torpedo-stage S7 embryo (Fig. 6a). After the S7 stage, the expression of MtABI3-like continued to increase as the cotyledons developed, seed filling, then desiccation occurred (Fig. 6b). The expression of *MtFUS3*-*like* associated with oil and protein accumulation continued after S7 and peaked about 15 days after anthesis (Fig. 6c).

### Group IV and Group V genes

**Fig. 7:**
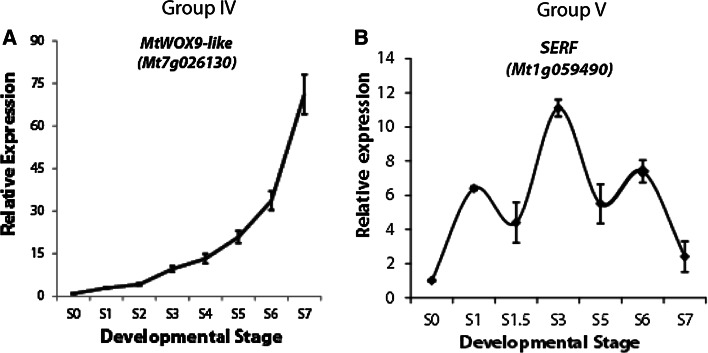
Expression of Group IV and V genes in ovule and embryo development. Expression profile of *MtWOX9*-*like* (**a**) and *MtSERF1* (**b**). Note *MtSERF1* data obtained from Mantiri et al. (2008). Supplementary Fig. S2 and redrawn, with approval (http://www.plantphysiol.org and Copyright American Society of Plant Biologists)

**Fig. 8:**
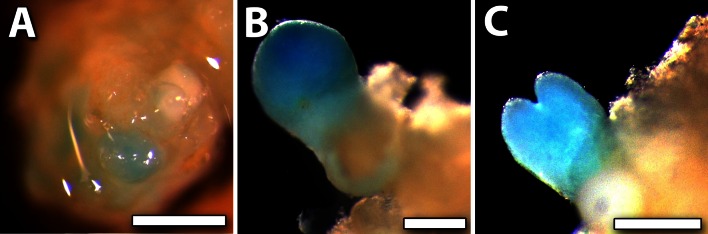
Localisation of *MtWOX9*-*like* expression. **a**–**c** GUS expression in somatic embryos driven by the *MtWOX9*-*like* promoter. **a** Embryo-specific expression in a proembryonic mass. **b** Globular-stage embryo. **c** Early torpedo-stage embryo. *Bars* 50 μm

In Group IV, there is a single gene (*MtWOX9*-*like*). This was the only gene that was progressively up-regulated during ovule and embryo development (S0-S7, Fig. 7a). To examine the localisation of *MtWOX9*-*like* expression, we created transgenic somatic embryos expressing a *MtWOX9*-*like* promoter-GUS fusion (*pMtWOX9*-*like::GUS*). Staining for GUS activity in this instance revealed a signal exclusively in somatic embryos and absent from the surrounding callus (Fig. 8a–c). The expression of *MtWOX9*-*like* localised throughout the embryo until at least the early torpedo stage (Fig. 8c).

In Group V, there is also a single gene (*MtSERF1*), This is the only gene which had a peak of expression at the S3 stage (Fig. 7b). *MtSERF1* is involved in somatic and zygotic embryogenesis in *M. truncatula* (Mantiri et al. 2008), and the data presented were obtained from this study.

## Discussion

In discussing the data, we examine the different transcriptional regulators in the context of ordered growth and development stages in early embryogenesis.

### Ovule development and early embryonic cell divisions (S0–S2)

The transcription factors examined show peak expression [Group I(A) and Group I(B)] or one peak of expression (Group II) during this phase. Two *WOX* genes and *MtWUS*, *MtDOF1*, five *HD*-*ZIP* genes (including *MtLMI1*-*like*) and one *KNOX* gene are in this category. Based on the data here and from the literature it is likely that *MtWOX 1*-*like, MtWOX4*-*like, MtDOF1*-*like* and *MtLMI1*-*l1ke* expression is associated with vascularisation; while *MtWUS, MtHD*-*ZIP2,3,4* and 5, and *MtKNOX7* expression is involved with ovule development and early embryonic cell divisions.

The *MtWOX4*-*like* and *MtWOX1*-*like* genes showed highest expression in pre-anthesis ovules (S0; Fig. 2a, b), decreasing during early embryogenesis. In Arabidopsis and tomato (*Solanum esculentum*), *WOX4* is involved in procambium development (Hirakawa et al. 2010; Ji et al. 2010). Similarly, *AtWOX1* is active in initiating the vascular primordium of Arabidopsis cotyledons (Haecker et al. 2004). In maize (*Zea mays*) embryos, *WOX4* marks the provasculature in hypocotyls (Nardmann et al. 2007). *MtWOX4*-*like* is strongly expressed in highly vascularised callus of *M. truncatula* wild-type Jemalong in response to auxin plus cytokinin (Chen and Rose, unpublished). The data at the SO-S2 developmental stage may reflect vascularisation of the funiculus in the developing ovule. Recent studies have identified *STENFOLIA* as the *MtWOX1*-*like* gene, which regulates leaf vascular patterning in *M. truncatula* and is also expressed at the base of ovules (Tadege et al. 2011, Table S3). The gene represented by TC161263, which we have called *MtDOF1*, is similar to *DOF4.6* from Arabidopsis (At4g24060). *MtDOF1* expression continually decreases during ovule development from stages S0–S6, but with increasing expression in torpedo-stage ovules (S7, Fig. 2h). In Arabidopsis *DOF4.6* plays a role, along with *AtHB*-*8*, in defining pre-procambium cell identity (Gardiner et al. 2010). *MtLM1*-*like* may also be involved in vascularisation (Fig. 4).

The expression of *MtWUS* in ovules isolated from flowers (S0 and S1) likely reflects an involvement in ovule development as occurs in Arabidopsis (Groß-Hardt et al. 2002), where it is expressed in the nucellus. Expression of *MtWUS* is still detectable in ovules from S2 pods, but not detected again until heart- and torpedo-stage embryos are formed (S6–S7 pods; Figs. 2i, 3a–c). In Arabidopsis, *WUS* expression appears in early globular (16-cell)-stage embryos (Mayer et al. 1998). Both qRT-PCR and in situ hybridisation demonstrate that in *M. truncatula*, *MtWUS* is not expressed at this stage in zygotic embryogenesis (Figs. 2i, 3a–c). Moreover, expression in heart-stage ovules (S6, Fig. 2i) remains low, as supported by in situ hybridisation (Fig. 3b, c). We compared the promoter regions (up to 4 kb upstream of the start codon) of *AtWUS* and *MtWUS*. Although we identified more than five conserved elements, the 57-bp spatial control region identified as essential for *AtWUS* promoter activity in the Arabidopsis stem cell niche (Baurle and Laux 2005) was absent from the *MtWUS* promoter. This difference may be related to the relative late expression in *Medicago* or the hypothesis that *MtWUS* is involved in the nodule meristem development as well (Couzigou et al. 2012).

Four *HOMEOBOX LEUCINE ZIPPER* (*HD*-*ZIP*) genes express similarly to *MtWOX4*-*like* and *MtWOX1*-*like* in the S0–S2 phases. The HD-ZIP class I and class II transcription factors have been associated with hormone-regulated cell growth and differentiation in response to environmental stimuli (Harris et al. 2011). Class I genes are involved in ABA responses and Class II genes in auxin responses. Both Class I (*MtHD*-*ZIP2* and *MtHD*-*ZIP4*) and Class II (*MtHD*-*ZIP3* and *MtHD*-*ZIP5*) genes have relatively high expression in the S0–S2 stages in the earliest stages of ovule and embryo development. A class I gene studied in *M. truncatula* (*MtHB1*, a homologue of *AtHB7* and *AtHB12*) is expressed in lateral root meristems induced by stress (Ariel et al. 2010). *MtHD*-*ZIP3* (Mt2g071640) in the MtGEA (Mtr.12147.1.S1_at probeset) has high expression in 2-week callus (Jemalong 2HA) when embryos are being initiated in response to auxin plus cytokinin. The class II *HD*-*ZIP2* gene represented by TC149707 and most closely related to *AtHB2* in Arabidopsis (At4g16780; Supplementary Fig. 1B) though having a similar expression to the other HD_ZIP gene in the S0–S6 stages shows increased expression in torpedo-stage ovules (S7; Fig. 2f).

Class I and Class II *KNOTTED1*-*like homeobox* (*KNOX*) genes are characterised by the presence of a homeodomain and MEINOX (also known as KNOX) domains (Mukherjee et al., 2009). *MtKNOX7* is a class II gene, and class II is less well studied. However, it is known that the MEINOX domain of class I KNOX genes can interact with other proteins such as BELL (Hay and Tsiantis 2010) to reduce GA levels (binds to promoters of *GA20ox1* and *GA2ox1*) and increase cytokinin levels (binds to promoter of *IPT7*). Such hormone influences favour cell division.

### Forming the globular-stage embryos (S3–S5)

The pattern of increasing *WOX9*-*like* expression during embryogenesis, as shown by qRT-PCR and also the GUS data in somatic embryos, is consistent with the maintenance of cell proliferation as the embryo develops. In Arabidopsis, *WOX9* is required for the maintenance of cell division in the embryo and suspensor (Wu et al. 2007). Importantly, *MtWOX9*-*like* in *Medicago* serves as a specific marker of somatic embryos that is active from the earliest stages of embryogenesis (Fig. 8a). Indeed, homologues of *MtWOX9*-*like* in *Brassica* *napus* (Malik et al. 2007) and *Vitis* *vinifera* (Gambino et al. 2011) have also been identified as excellent markers of somatic embryogenesis. Wu et al. (2007) also showed that *WOX8* and *WOX2* act redundantly to promote cell division in Arabidopsis, but with a minor role compared to *WOX9*.

Apart from the *MtWOX9*-*like* gene, the only other gene showing increasing expression in the S2–S5 stages is *MtSERF1*, while *MtSERF1* is essential for somatic embryogenesis its function is not known. Previous in situ hybridisation studies have shown expression throughout globular-stage embryos and localisation in the organiser region in the heart-stage embryo (Mantiri et al. 2008). This suggests that it may be related to regulating the growth of the globular-stage embryo and the development of the organiser region, though more work is necessary. *SERF1* is ethylene dependent in somatic embryogenesis (Mantiri et al. 2008; Zheng et al. 2013). In *Medicago*, *MtSERF1* is dependent on auxin, cytokinin and ethylene (Mantiri et al. 2008).

### Heart and torpedo stage and formation of apical meristems (S6–S7)

In the heart stage zygotic embryo of *M. truncatula, MtWUS* expression has previously been shown to localise to the organiser region of the shoot pole of the heart-stage embryo (Chen et al. 2009). In contrast to Arabidopsis where *WUS* expression first appears in early globular (16-cell)-stage embryos (Mayer et al. 1998), there is no expression at this stage in *M. truncatula*. Interestingly, *WUS* expression in embryogenic cultures of Arabidopsis is induced by auxin in Arabidopsis, but by cytokinin in *M. truncatula* (Chen et al. 2009; Su et al. 2009). The primary amino acid sequence and domain structure of WUS is conserved between Arabidopsis and *M. truncatula*. As discussed above, the 57-bp spatial control region identified as essential for *AtWUS* promoter activity in the Arabidopsis stem cell niche (Baurle and Laux 2005) was absent from the *MtWUS* promoter.

The expression pattern of *MtWOX5* is similar to *MtWUS* in the S3–S7 stages (Fig. 2i, m), and both genes participate in the formation of apical meristems at different poles of the embryo (Chen et al. 2009). Similar to *MtWUS*, expression of *MtWOX5* in *Medicago* occurs later in embryogenesis relative to its Arabidopsis homologue (Haecker et al. 2004). It may be that before the heart stage, the actual establishment of stem cell niches in *Medicago* is performed by additional genes.

The *M. truncatula* homologue of Arabidopsis *LATE MERISTEM IDENTITY1* (*MtLMI1*-*like*) encodes a Class I HD-ZIP protein with an expression profile that closely resembles *MtWUS* (Fig. 2i, j). These two genes have two common promoter elements which suggest common targets for regulatory proteins to facilitate co-expression. RNA in situ hybridisation shows that unlike *MtWUS*, expression of *MtLMI1*-*like* localises throughout the whole embryo at the torpedo stage with no expression in the hypophysis and suspensor, but with a concentration of signal in the forming vascular tissue (Fig. 4b, c). The Arabidopsis homologue promotes floral meristem identity (Saddic et al. 2006); but the Arabidopsis HD-ZIP protein AtHB-8 is associated with procambium development (Gardiner et al. 2010); while a HD-ZIP from tomato, VAHOX1, is involved in phloem development during secondary vasculature formation (Tornero et al. 1996).

Members of the *KNOX* gene family are necessary for maintaining the population of stem cells in the shoot apical meristem. *SHOOT MERISTEMLESS* (*STM*) functions to maintain central meristem cells in an undifferentiated state (Endrizzi et al. 1996) and has a complementary role to *WUS* in Arabidopsis shoot meristem regulation (Lenhard et al. 2002). The Class I *MtKNOX6* gene (Mt5g093180) is *STM*-*like* (Di Giacomo et al. 2008) and has an expression profile similar to *MtWUS* (Fig. 3i, k). The Class II *MtKNOX4* gene (Mt5g011210) showed increased expression in torpedo-stage (S7) ovules and had a similar expression pattern to *MtKNOX6* (Fig. 2k, l). It is likely that MtKNOX4 and 6 play roles in shoot meristem formation in embryogenesis. MtWUS, MtWOX5, MtMLI1-like and MtKNOX6 (Fig. 5d) have common upstream GAGA elements, recognised by regulatory proteins in soybean (Sangwan and O’Brian 2002).

The expression profile of *MtWOX11*-*like* closely resembles that of *MtWOX5*, increasing in expression only during the latter stages of embryogenesis (S6–S7; Fig. 2n). Similarly, the grapevine homologue of *MtWOX11*-*like* is up-regulated only in the later stages of somatic embryogenesis (Gambino et al. 2011). A homologue in rice (Os07g48560 in Supplementary Fig. 1A) is expressed in both shoot and root apical meristems and is involved in adventitious root formation (Zhao et al. 2009). Thus, *MtWOX11*-*like* might participate in the regulation of meristem formation during late embryogenesis in *M. truncatula.*

The *BABYBOOM* (*MtBBM*) gene belongs to the APETALA2 (AP2) family of transcription factors. *BBM* in Arabidopsis was so-named for its capacity to induce somatic embryogenesis when ectopically expressed (Boutilier et al. 2002). *MtBBM* is strongly up-regulated in torpedo-stage ovules (S7; Fig. 2p). In vitro, *MtBBM* is also up-regulated in root primordia (Imin et al. 2007) and may be involved in the establishment of the root apical meristem. *MtBBM* and *MtFUS3*-*like* genes exhibited a similar expression profile and both genes contain common motifs in their 3′-UTR as determined by Mclip (Fig. 6b). While *MtBBM* and *MtFUS3*-*like* have different cellular functions there is a substantive development of the embryonic root in legumes which also accumulates storage products (Wang et al. 2012).

### The embryo maturation phase

The B3 domain-containing transcription factor family includes *FUSCA3* (*FUS3*) and *ABA INSENSITIVE3* (*ABI3*), which are involved in embryogenesis and seed filling (Giraudat et al.^1992^; Luerssen et al. 1998). *MtFUS3*-*like* and *MtABI3*-*like* both have high expression at the torpedo stage and negligible earlier expression, although *MtFUS3*-*like* expression starts slightly earlier (Fig. 2o, p). What is of interest is how early in embryogenesis the expression of the seed filling-related transcription factors initiate expression. *MtABI3*-*like* expression occurs throughout torpedo-stage embryos, but not in the hypophysis and suspensor. The expression of these two genes continues through the seed-filling stage (Thompson et al. 2009), but with *MtFUS3*-like expression declining earlier (Fig. 6).

Maximising the amount of storage product per seed is a combination of the size of the embryo and the amount of packaged storage product in each cell. Therefore, transcription activity in the S3–S5 stage of genes such as *MtWOX 9*-*like* and *MtSERF1* may be particularly important in affecting embryo size, while genes such as MtABI3 and MtFUS3 expressed subsequently influence the amount of storage product in each cell.

## Conclusions

We developed a method of examining highly standardised stages of zygotic embryo development in *M. truncatula*. This enabled us to establish expression patterns of candidate transcription factors and their potential nexus. We presented data for 19 transcription factors that changed their expression during ovule formation and early embryogenesis. Transcription factors likely to play roles in ovule development, very early embryogenesis, mid-stage development and the development of the apical meristems were described. Expression of other transcription factors was associated with vascularisation and the onset of cotyledon development and seed storage. What is also of interest is how a number of transcription factors have peaks of expression in ovule and early embryo development as well as the torpedo stage. In some cases, co-expression of some genes could be related to common regulatory sequences in the promoter or 3′-UTR regions. Identifying stage-specific regulators is of great importance in providing approaches for functional studies. Maximising the amount of storage product per seed is a combination of the size of the embryo and the amount of packaged storage product in each cell. The genetic regulation of embryogenesis throughout all developmental stages needs to be understood to maximise the yields of storage products in legumes.

## Electronic supplementary material

Below is the link to the electronic supplementary material.

Supplementary material 1 (DOCX 575 kb)

## Abbreviations

HD-ZIP: HOMEOBOX LEUCINE ZIPPER
KNOX: KNOTTED-LIKE HOMEOBOX
MtGEA: *Medicago truncatula* Gene Expression Atlas
WOX: WUSCHEL-related homeobox
WUS: WUSCHEL
