# Supplementary material for: Transcriptional regulation of early embryo development in the model legume Medicago truncatula
Source: Plant Cell Rep. 2013 Nov 22;33(2):349–62. doi: 10.1007/s00299-013-1535-x (PMC3909251; doi:10.1007/s00299-013-1535-x)
Supplement: Supplementary file 1 — Supplementary material 1 (DOCX 575 kb) [file 299_2013_1535_MOESM1_ESM.docx]

**Table S1:** **Time course for pod collection (time from previous stage)**

|  | Flower Stage 1 (S0) | Flower Stage 2 (S1) | Pod Stage 1 (S2) | Pod Stage 2 (S3) | Pod Stage 3 (S4) | Pod Stage 4 (S5) | Pod Stage 5 (S6) | Pod Stage 6 (S7) |
| --- | --- | --- | --- | --- | --- | --- | --- | --- |
| Days | 0 | 2 | 1.5-2 | 0.5-1 | 0.5 | 0.5-1 | 1 | 1-1.5 |

**Table S2:** **Sequences of primers used in qPCR**

| Gene Name | Primer Sequences |
| --- | --- |
| *MtWOX1-like* | AACTGGCTACCATCTACAAATTCCA |
|  | TCTTCTTTGCTGTAATTCTGCTTCA |
| *MtWOX4-like* | GTTTCATTAGACCCGAAACTGCTC |
|  | TTGTTCTTGTGTTGGATTCCATCTT |
| *MtWOX5* | TATGAATGCTTCAACACAAAGGTT |
|  | CCAAATGAGTTCAATGGAAAAAGC |
| *MtWOX9-like* | TCCCCATTATTGATCATCGACAT |
|  | CAAAGGATGCACGAACGTTAAAG |
| *MtWOX11-like* | AAATTCAAGCAGGGCCACTAGAC |
|  | GGTGCAAGCTCTGAATCAAGATG |
| *MtWUS* | CTTACAACATTTCATCTGCTGGGCT |
|  | CGACATGATGACCAATCCATCCTAT |
| *MtLMI1-like* | GCGATTAACGAGTGAACAGATGG |
|  | GCTGTAGCCCTAACTCCTTGGAA |
| *HD-ZIP2 (AtHB2-like)* | GTCGAAGGAGCAGTCAATGTTGT |
|  | CTTGGCTTCAGATTCAACTGCTT |
| *HD-ZIP3 (HAT14-like)* | TGCAACAAATTCTACAGCCACTTC |
|  | TTTGAGGTGGAATCATCACTGGT |
| *HD-ZIP4 (AtHB13-like)* | CAGAACCAAAAACTTCAAGCTGAGA |
|  | TGATCCTTCTGTTTCTTTGTTCAGG |
| *HD-ZIP5 (HAT9-like)* | TCTAAGGCCACGACAAGTTGAAG |
|  | TTTTCAAGAACTCGCAATCCACCT |
| *MtKNOX6 (STM-like)* | GACAACAACTACTTGATTGGTGGAG |
|  | CTTCAAATCTAGGCCTGTTGATTCT |
| *MtKNOX4 (KNAT7-like)* | CAAACTCATTCTCTTTCGCCTCAT |
|  | ACAGCTTCAACAGCATGAACTCTG |
| *MtKNOX7 (KNAT4-like)* | CGCATTATGTTCTCCTGCTCTGT |
|  | CGGTCAAGCTTTGTAGAGACTGC |
| *MtSERF* | TCATACGCCATCATCTCTTAGGT |
|  | AGGGGTTGTTTCCTTTGAAGAT |
| *MtABI3-like* | TGCAGACACAGAGTTCTCACCAG |
|  | GACTTCCGACATCACTTTGCTTC |
| *MtFUS3-like* | GCTTTCCTTCCTGTCCTTGAGTC |
|  | GATCACAAGCTTTCTTTGCCTGA |
| *MtBBM* | GATCTCTACCTAGGAACTTTCAGCAC |
|  | TGTCAAAGTTTGTAACTGCACTCAG |
| *MtDOF1* | ACCATAAGATCCATCATGAAGAAAG |
|  | AGAAGTGTTGTTATTTTGCTGAACC |
| *MtGAPDH* | GACTTTATTGGTGATACCAGGTCG |
|  | GGTCAACCACACGGGTACTGTAA |

**Table S3:** **Gene Loci, Arabidopsis homologues and Affymetrix Probesets**

| Gene Name | Medicago Locus^1^  ^Mt3.5^ | Medicago Locus^2^  ^Mt3.0 Phytozome 9.0^ | Closest Matching Arabidopsis Locus; Name^3^; identity of amino acids | Affymetrix *M. truncatula* Probeset^3^ | Tissue-specific expression in *M. truncatula* gene atlas | Functional / developmental studies in *M. truncatula* |
| --- | --- | --- | --- | --- | --- | --- |
| *MtWOX1-like* | Medtr4g154980 | Medtr8g107210 | At3g18010; *WOX*; 38.7% | Mtr.51678.1.S1_at | Flowers,vegetative buds and seed maturation | Blade outgrowth and leaf vascular patterning in Medicago (Tadege et al. 2011) |
| *MtWOX4-like* | Medtr1g024140 | Medtr1g019130 | At1g46480; *WOX4*; 55.3% | Mtr.23994.1.S1_at | Flowers, shoots and stems | In vitro root formation – low expression (Imin et al. 2007) |
| *MtWOX5* | Medtr5g089330 | Medtr5g081990 | At3g11260; *WOX5*; 55.4% | Mtr.33304.1.S1_at | Leaf tissue culture during first two wks | Root meristem development (Chen et al. 2009) |
| *MtWOX9-likeB*^6^ | Medtr7g026130 | Medtr7g025010 | At2g33880; *WOX9*; 36.9% | Mtr.5935.1.S1_at | NA | NA |
| *MtWOX11-like* | Medtr7g103730 | Medtr7g086940 | At3g03660, *WOX11*; 45.9% | Mtr.28819.1.S1_at | Flowers, seed coat, seed development | NA |
| *MtWUS* | Medtr5g021830 | Medtr5g021930 | At2g17950, *WUS*; 39.0% | NA | NA | Somatic and zygotic embryogenesis (Chen et al. 2009) |
| *MtLMI1-like* | Medtr1g073710 | Medtr1g061660 | At5g03790; *LMI1*; 44.5% | Mtr.18411.1.S1_s_at | Vegetative bud and seed | NA |
| *HD-ZIP2 (AtHB2-like)* | NA (TC149707) | NA | At4g16780; *ATHB2* | Mtr.11045.1.S1_at | Seed development | NA |
| *HD-ZIP3 (HAT14-like)* | Medtr2g071640 | Medtr2g061030 | At5g06710; *HAT14*; 45.0% | Mtr.12147.1.S1_at | Leaf tissue culture in first 2 wks | NA |
| *HD-ZIP4 (AtHB13-like)* | Medtr5g040020 | Medtr5g039000 | At1g69780; *ATHB13*; 61.7% | Mtr.10964.1.S1_at | Hypocotyl | NA |
| *HD-ZIP5 (HAT9-like)* | Medtr5g015030 | Medtr5g014890 | At2g22800; *HAT9*; 60.1% | Mtr.40459.1.S1_at | Stem | NA |
| *MtKNOX6 (STM-like)* | Medtr5g093180 | Medtr5g085860 | At1g62360; *STM*; 49.1% | Mtr.32410.1.S1_at | Most highly in stems, but also in seed filling | Shoot apical meristem (Giacomo et al. 2008) |
| *MtKNOX4 (KNAT7-like)* | Medtr5g011210 | Medtr5g011070 | At1g62990; *KNAT7;* 74.4% | Mtr.38132.1.S1_a_at^5^ | Highly in seed coat, roots, also seed maturation | Shoot apical meristem (Giacomo et al. 2008) |
| *MtKNOX7 (KNAT4-like)* | NA (TC149968 ) | NA | At5g11060; *KNAT4* | Mtr.8750.1.S1_at | Expressed widely, but highest in nodules | Shoot apical meristem (Giacomo et al. 2008 |
| *MtSERF1* | Medtr1g059490 | Medtr1g040430 | AT5G61590; NA; 42.6% | Mtr.38122.1.S1_at | Embryo | Somatic and zygotic embryogenesis (Mantiri et al. 2008) |
| *MtABI3-like* | NA (TC162720) | NA | At3g24650; *ABI3* | Mtr.44550.1.S1_at | Seed-specific | Seed filling (Verdier et al. 2008) |
| *MtFUS3-like* | Medtr7g096720 | Medtr7g083700 | At3g26790; *FUS3*; 47.3% | Mtr.25862.1.S1_at | Seed-specific | Seed filling (Verdier et al. 2008) |
| *MtBBM* | Medtr7g090100 | Medtr7g080460 | At5g17430; *BBM;* 45.0% | Mtr.21627.1.S1_at | Leaf tissue culture, seed and root | In vitro root formation – high expression (Imin et al. 2007) |
| *MtDOF1* | NA (TC161263) | NA | At4g24060; *DOF4.6* | Mtr.45367.1.S1_at | Expressed widely | NA |
| *MtGAPDH* | Medtr8g109660 | Medtr4g103920 | At3g04120; *GAPC;* 87.6% | Mtr.51186.1.S1_at | Expressed everywhere at a similar level | NA |
| ^1^Obtained from the Mt3.5 genome release (http://www.medicagohapmap.org) or, where no data exist, a corresponding TC sequence from the *M. truncatula* Gene Index is given (http://compbio.dfci.harvard.edu/tgi). ^2^ Mt 3.0 genome release (http://www.medicagohapmap.org) used in Phytozome 9.0. ^3^Based on phylogenetic analysis of protein sequences or where no full length sequence was available by BLAST searches. ^4^Only the best matching probeset (E-value = 0) from the Affymetrix *M. truncatula* GeneChip shown. ^5^Probeset match has a large E-value (1x10^-56^). ^6^ We obtained the full length coding sequence from cDNA to correctly annotate this gene locus on Phytozome. There are two WOX9-like genes (Medtr2g015000 and Medtr7g025010; *MtWOX9-likeA and B* respectivel*y*) and both with closer identity to *AtWOX9* than *AtWOX8*. The amino acid identity between *MtWOX9-likeB* and *AtWOX8* is less than between *MtWOX9-likeA* and AtWOX8). NA; no data available. | | | | | | |


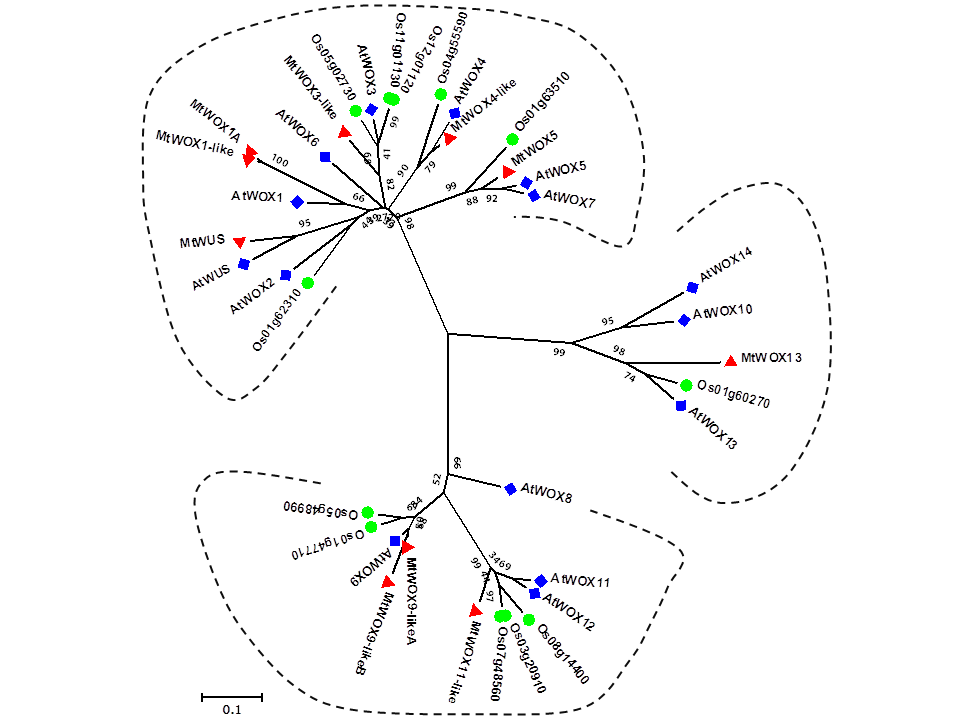


**Supplementary Fig. 1A.** Phylogenetic tree of *WOX* genes from Arabidopsis, Medicago and rice. Dendrogram is based on full-length amino acid sequences. Bootstrap values are expressed as percentage (1000 rounds). Putative *M. truncatula* orthologues to Arabidopsis proteins examined in this study are shown in red.


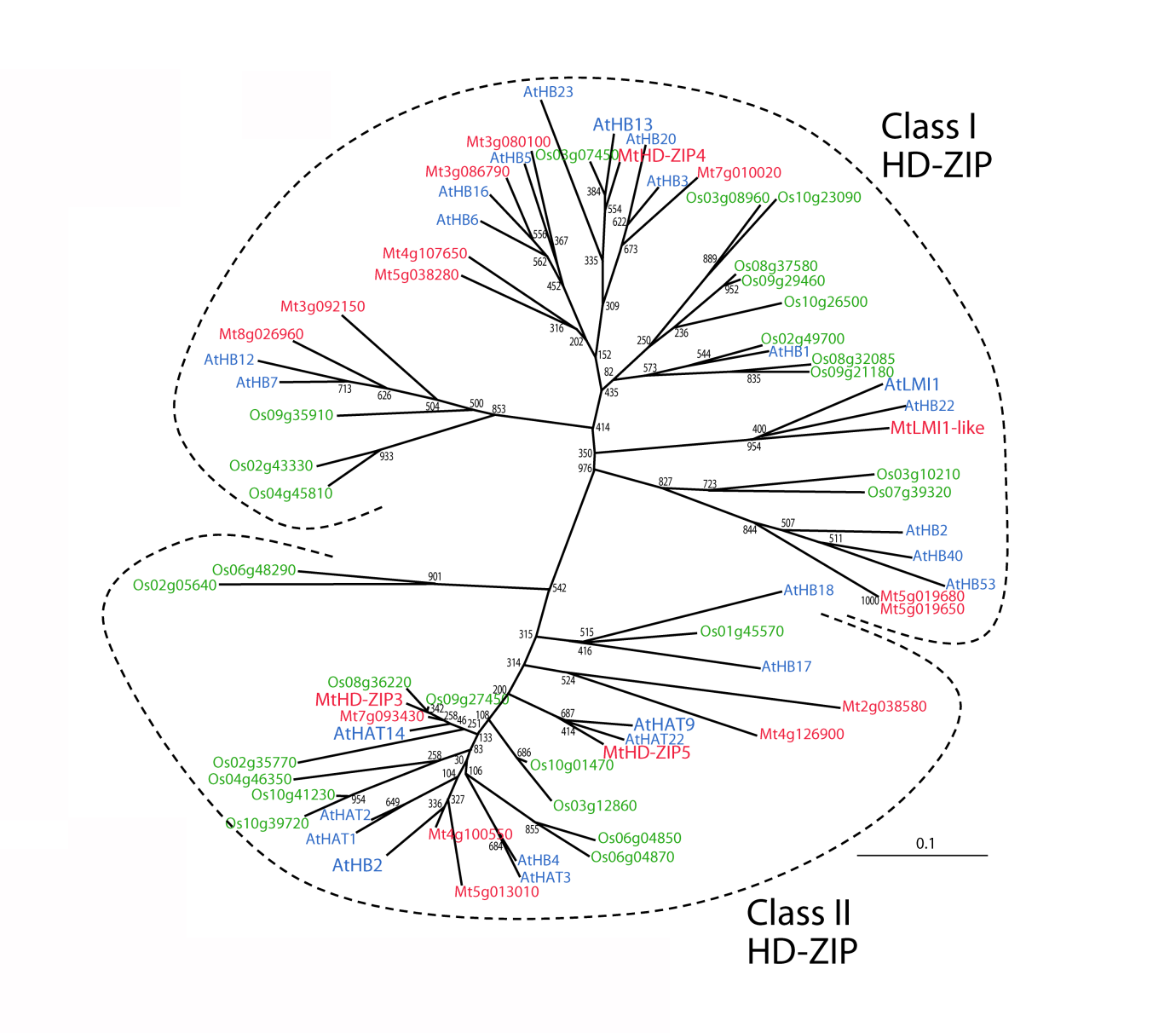


**Supplementary Fig. 1B.** Phylogenetic tree of HD-ZIP genes from Arabidopsis, Medicago and rice. Dendrogram is based on full-length amino acid sequences. Bootstrap values are for 1000 rounds. Putative *M. truncatula* orthologues to Arabidopsis proteins examined in this study are shown in red.


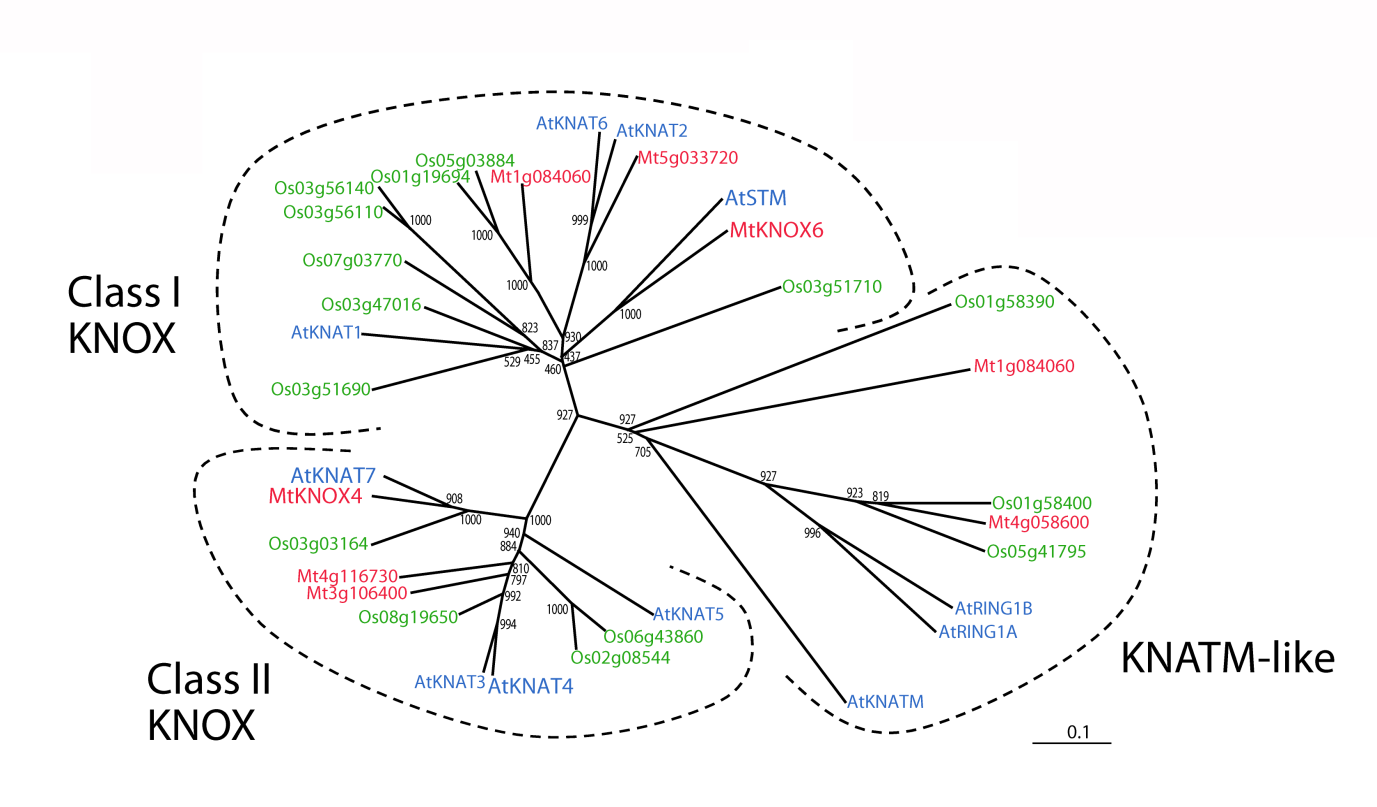


**Supplementary Fig. 1C.** Phylogenetic tree of HD-ZIP genes from Arabidopsis, Medicago and rice. Dendrogram is based on full-length amino acid sequences. Bootstrap values are for 1000 rounds. Putative *M. truncatula* orthologues to Arabidopsis proteins examined in this study are shown in red.
